# Original Hosts, Clinical Features, Transmission Routes, and Vaccine Development for Coronavirus Disease (COVID-19)

**DOI:** 10.3389/fmed.2021.702066

**Published:** 2021-07-06

**Authors:** Ting Wu, Shuntong Kang, Wenyao Peng, Chenzhe Zuo, Yuhao Zhu, Liangyu Pan, Keyun Fu, Yaxian You, Xinyuan Yang, Xuan Luo, Liping Jiang, Meichun Deng

**Affiliations:** ^1^Department of Biochemistry and Molecular Biology, Hunan Province Key Laboratory of Basic and Applied Hematology, School of Life Sciences, Central South University, Changsha, China; ^2^Department of Cardiovascular Medicine, The Third Xiangya Hospital, Central South University, Changsha, China; ^3^Hunan Key Laboratory of Animal Models for Human Diseases, Hunan Key Laboratory of Medical Genetics, School of Life Sciences, Central South University, Changsha, China; ^4^Xiangya School of Medicine, Central South University, Changsha, China; ^5^Hunan Yuanpin Cell Biotechnology Co., Ltd, Changsha, China

**Keywords:** SARS-CoV-2, COVID-19, original host, transmission modes, vaccine development

## Abstract

The pandemic of coronavirus disease 2019 (COVID-19), which is caused by severe acute respiratory syndrome coronavirus 2 (SARS-CoV-2), has led to public concern worldwide. Although a variety of hypotheses about the hosts of SARS-CoV-2 have been proposed, an exact conclusion has not yet been reached. Initial clinical manifestations associated with COVID-19 are similar to those of other acute respiratory infections, leading to misdiagnoses and resulting in the outbreak at the early stage. SARS-CoV-2 is predominantly spread by droplet transmission and close contact; the possibilities of fecal–oral, vertical, and aerosol transmission have not yet been fully confirmed or rejected. Besides, COVID-19 cases have been reported within communities, households, and nosocomial settings through contact with confirmed COVID-19 patients or asymptomatic individuals. Environmental contamination is also a major driver for the COVID-19 pandemic. Considering the absence of specific treatment for COVID-19, it is urgent to decrease the risk of transmission and take preventive measures to control the spread of the virus. In this review, we summarize the latest available data on the potential hosts, entry receptors, clinical features, and risk factors of COVID-19 and transmission routes of SARS-CoV-2, and we present the data about development of vaccines.

## Introduction

In late December 2019, a novel coronavirus associated with pneumonia spread rampantly. The World Health Organization (WHO) named the infectious condition coronavirus disease 2019 (COVID-19), and the virus was classified as severe acute respiratory syndrome coronavirus 2 (SARS-CoV-2). Compared to SARS-CoV-1, SARS-CoV-2 has a higher basic reproduction number and higher transmissibility. The origin of SARS-CoV-2 remains unknown and within a short period, COVID-19 has become a serious threat to the global economy and human health. The clinical symptoms generally include fever, fatigue, cough, vomit, diarrhea, and dyspnea in humans (1). However, patients in the incubation period and asymptomatic patients do not show these symptoms, and are hence easily misdiagnosed, which leads to an increased risk of SARS-CoV-2 transmission. It has been reported that each patient with COVID-19 infects ~2.2 close contacts (2, 3). Currently, there is no specific treatment for COVID-19, so the best strategy to control the number of COVID-19 cases is to limit the spread of the virus. To predict the epidemic trend and guide control measures, reliable information is urgently needed. In this review, we summarize the recent data on potential SARS-CoV-2 hosts, the clinical features of COVID-19, the risk factors of severe COVID-19, and the different modes of viral transmission. Finally, we summarized the safety and efficacy of some vaccines.

## Potential Hosts of SARS-CoV-2

**Figure 1:**
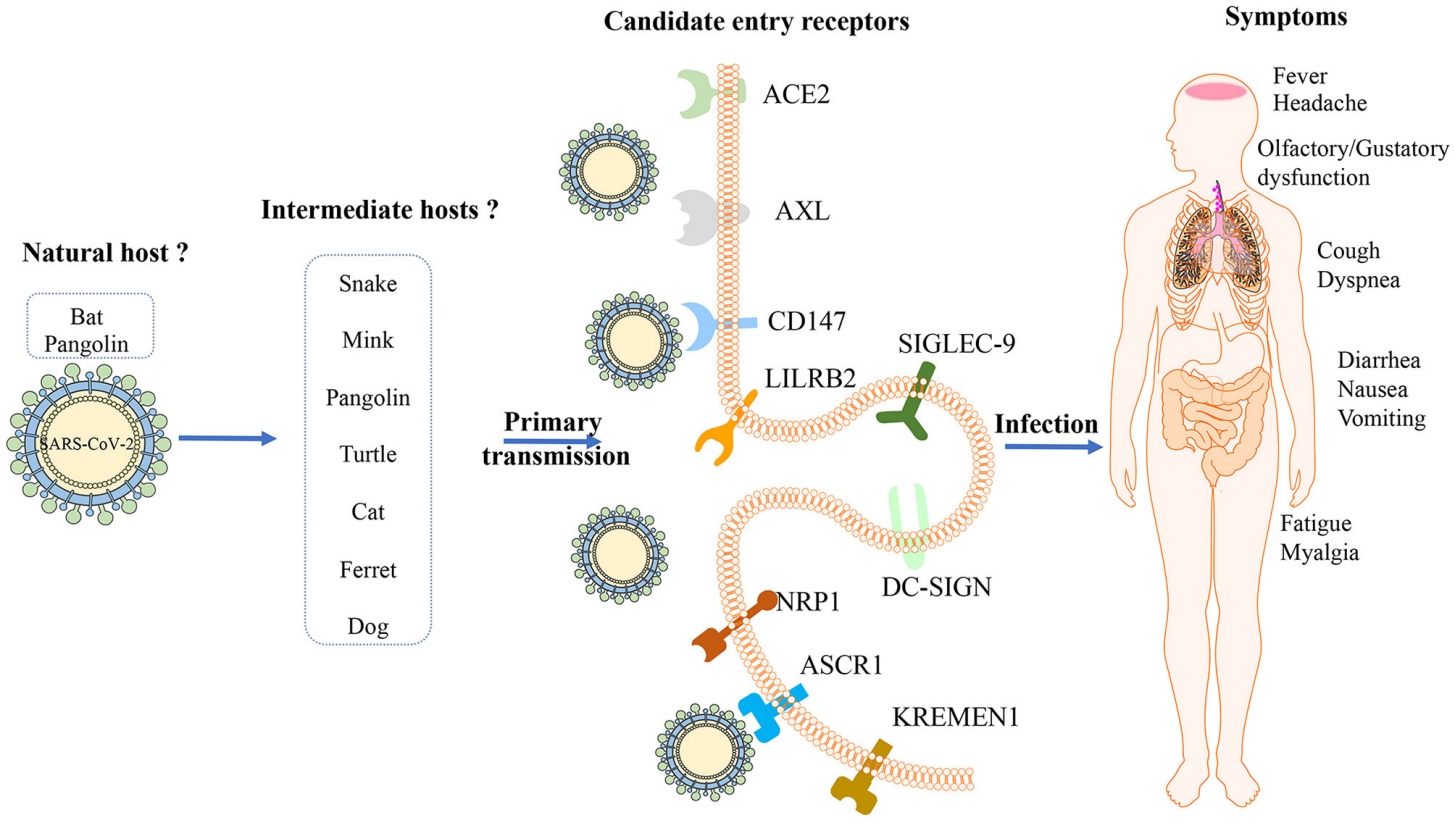
Potential hosts, entry receptors and clinical features of SARS-CoV-2. Bats might be the original host of ARS-CoV-2 and several animals including snake, mink, pangolin, turtle, cat, ferret and dog might be intermediate hosts; Several receptors, including ACE2, AXL, CD147, LILRB2, SIGLEC-9, DC-SIGN, NRP1, ASCR1, and KREMEN1, have been recognized as candidate cell entry receptors for SARS-CoV-2. The initial manifestations associated with COVID-19 are not specific, including fever, cough, fatigue, myalgia, and dyspnea, which could involve in several organs.

Virus hosts are divided into natural, intermediate, and final hosts according to the viral transmission routes and the progenitor viruses existing in the natural host cannot effectively use human susceptible cell receptors and fail to invade directly, which is the main factor that limits the direct transmission of progenitor viruses to humans (4). Despite the international research effort conducted, a natural host, either direct or intermediate, has not yet been identified (Figure 1). It is currently thought that SARS-CoV-2 has a zoonotic origin and has secondarily acquired human-to-human spreading capacity (5).

The discovery of diverse bat coronaviruses closely related to SARS-CoV-2 suggests that bats are possible reservoirs of SARS- CoV-2 (6). Bats are critical natural hosts of alpha- and beta-coronavirus. So far, the virus closest to SARS-CoV-2 is a bat coronavirus named RaTG13 found in *Rhinolophus affinis* in Yunnan Province, China, whose full-length genome sequence is 96.2% matching the total length of SARS-CoV-2 (7) and phylogenetic analysis verified that SARS-CoV-2 is tightly combined with RaTG13 (8). Another novel bat virus RmYN02 noted more recently in a *Rhinolophus malayanus* bat found in Yunnan is 93.3% identical to SARS- CoV-2 across the genome, which exhibits 97.2% identity to SARS- CoV-2, which is even higher than for RaTG13 in the long lab gene (9). Moreover, bat related coronaviruses ZC45 and ZXC21, which are previously detected in *Rhinolophus pusillus* bats in eastern China, also belong to the SARS-CoV-2 lineage of the sarbecovirus subgenus by phylogenetic analysis (10).

From 2017 to 2019, a variety of SARS-CoV-2 related viruses have been identified in *Malayan pangolin* tissues smuggled from Southeast Asia to China. Some independent studies suggested the pangolin as SARS-CoV-2 host (11–13), while other studies proposed that the pangolin may be a natural host rather than an intermediate host (14, 15). The latest study revealed the receptor-binding domain (RBD) of the S protein of Guangdong pangolin coronaviruses is similar to SARS-CoV-2 with one difference in a non-critical amino acid, remaining all five critical residues identical for receptor binding (12). Compared with the Guangdong strains, the pangolin coronaviruses reported in Guangxi is similar to SARS-CoV-2 with only 85.5% genome sequence identity (11). The results indicate that pangolins have the potential to act as intermediate hosts for SARS-CoV-2, but the genome identity of pangolin coronaviruses known to date with SARS-CoV-2 does not exceed 92% (16). Existing data are insufficient to be the evidence explaining that pangolins directly participated in the emergence of SARS-CoV-2.

Additionally, comprehensive sequence analysis and comparison with respect to relative synonymous codon usage (RSCU) suggested that SARS-CoV-2 has a codon usage bias similar to that in snakes (17). However, it's argued that the data based on RSCU were not sufficient to regard snakes as an immediate host of SARS-CoV-2, as the relation between coronaviruses and vertebrates has never been reported (14). By predicting the interaction between the RBD of coronavirus spike protein and the host receptor, angiotensin-converting enzyme 2 (ACE2) with systematic comparison and analysis, turtles (*Chrysemys picta bellii, Chelonia mydas*, and *Pelodiscus sinensis*) should also be considered as potential intermediate hosts that can transmit SARS-CoV-2 to humans (18).

Besides wildlife, a virus–host prediction analysis with a deep learning algorithm showed that the infectivity pattern of potential hosts by mink viruses resembles that of SARS-CoV-2 (19), which is subsequently confirmed by a report on the outbreak of SARS-CoV-2 infection in farmed mink documenting the susceptibility of mink in Netherlands (20). Although most infected minks have mild symptoms, some develop severe respiratory distress and die of interstitial pneumonia. But the latest test of mink farm related staff showed 68% had evidence of SARS-CoV-2 infection with an animal sequence signature, which proved the two-way transmission on mink farms (21). A serological investigation evaluated the infection of cats by SARS-CoV-2 by detecting specific neutralizing serum antibodies (22), and it demonstrated that SARS-CoV-2 could infect cat populations. But cats will recover quickly after being infected with SARS-CoV-2, and their infectiousness will not last too long, so cats will not pose a threat to human health in the long run and the potential for the spread of SARS-CoV-2 from cats to humans can't be ignored. Meanwhile, 2 out of 15 dogs from households with confirmed human cases of COVID-19 in Hong Kong were found SARS-CoV-2 positive and remained asymptomatic during quarantine (23). An experimental study showed that SARS-CoV-2 effectively replicates in the upper respiratory tract of cats and ferrets, while dogs, pigs, chickens and ducks are not sensitive to SARS-CoV-2 (24). So far, studies based on RBD domain analysis have ruled out the possibility of mice, rats and rabbits participating in the SARS-CoV-2 cycle (25). These results suggest that people with COVID-19 should limit contact with their pets to avoid potential transmission.

It is worth noting that investigations and researches deny that SARS-CoV-2 emerged through laboratory manipulation of any linked SARS-CoV-like coronavirus and for the RBD of SARS-CoV-2 is optimized and can be combined with human ACE2 through an effective solution different from the previously predicted solution (5). In addition, if genetic manipulations have been performed, one of several reverse gene systems available for beta-coronavirus may have been used, the genetic data irrefutably demonstrate that SARS-CoV-2 is not derived from any previously used virus backbone (26). But on the basis of other analyses, an artificial origin of SARS-CoV-2 is not a baseless conspiracy theory that is to be condemned (27, 28). They presume that SARS-CoV-2 may be a chimera, most of its sequence is closest to bat coronavirus RaTG13, and its RBD is almost the same as pangolin MP789-like CoV, and the furin cleavage site in the SARS-CoV-2 spike protein which gives the virus the ability to cross species and tissue barriers was previously not seen in other SARS-like CoVs, thus human intervention cannot be ruled out. However, this hypothesis was immediately rejected in the view that SARS-CoV-2 origin doesn't require recombination. The RBD of SARS-CoV2 represent a non-recombinant variant of the ancestor and the hypothesis of artificial creation does not agree with a number of findings based on genetic analysis of SARS-CoV-2 and its relatives (29). The origin of SARS-CoV-2 is the subject of many hypotheses, most arguments have greatly weakened the hypothesis of the laboratory origin, and the hypothesis of natural origin is consistent with all available genetic and experimental data.

The source of SARS-CoV-2 remains increasingly obscure, but it is crucial to determine where exactly it first appeared and how it initially spread through the population to clarify the viral transmission routes and to eliminate secondary transmission. Hence, it is necessary to strengthen cooperation between countries to treat patients and conduct COVID-19-related research to overcome the novel coronavirus pandemic.

## Entry Receptor for SARS-CoV-2

Attachment of the viruses to the cell surface receptor is the first step in infection and most viruses have evolved to recognize receptors, which are glycans on cell surface glycoproteins or glycolipids (30). After the SARS-CoV outbreak in 2003, ACE2 was confirmed as a receptor that enters lung epithelial cells (Figure 1) (31). It seems that both SARS-CoV-2 and SARS-CoV use a similar host cell entry mechanism and work by binding to the host ACE2, which is located on the surface of the host cell and is abundantly present (32). ACE2 is a type I transmembrane protein, which is mainly involved in the regulation of blood pressure, humoral balance and cell proliferation. It consists of a highly glycosylated N-terminal domain located outside the cell and a shorter C-terminal domain located in the cell. The N-terminal domain contains the binding site of the virus S protein. ACE2 is mainly distributed in alveolar epithelial cells, intestinal epithelial cells and bronchial epithelial cells. Some studies have shown that it is also expressed in vascular endothelial cells, heart, kidney and other organs, but the expression is low in spleen, thymus, lymph nodes, bone marrow and immune cells (33). The results of pathological study also showed that the pathological damage caused by SARS-CoV-2 was mainly concentrated in the lung, and the digestive organs and kidneys were also damaged to varying degrees (34). It's worth noting that the affinity between ACE2 and a SARS-CoV-2 S ectodomain is ~10–20-fold higher than ACE2 and SARS-CoV S (35). To fulfill its entry, the SARS-CoV-2 spike binds to its receptor human ACE2 (hACE2) through its RBD and is activated by human proteases proteolytically. It shows although SARS-CoV-2 RBD albeits more potent, its exposure is less than SARS-CoV RBD. And SARS-CoV-2 is preactivated by the proprotein convertase furin, thereby reducing its dependence on the entry of target cell proteases, which is different from SRAR-CoV (36).

Relying on ACE2 receptor alone does not seem to be enough to explain the strong infectivity and transmission of SARS-CoV-2. Although the role of ACE2 as a SARS-CoV-2 receptor is clear, studies have shown that the expression of ACE2 in various human tissues, especially in the respiratory tract, is extremely low. Combining proteomics, bioinformatics and computational biology methods, researchers found that the tyrosine-protein kinase receptor UFO (AXL) interacts with the N-terminal domain of SARS-CoV-2 S specifically and there is a strong co-localization on the cell membrane. In the bronchoalveolar lavage fluid cells of COVID-19 patients, the expression level of AXL is highly correlated with the level of SARS-CoV-2 S and AXL is highly expressed in almost all types of respiratory system cells including lung type I/II epithelial cells, basal cells, and fibroblasts. So AXL is a candidate receptor for SARS-CoV-2 (37). Additionally, CD147 is a transmembrane glycoprotein, a member of the immunoglobulin superfamily, involved in tumorigenesis and development, malaria parasite invasion and influenza virus infection. CD147 exists in a variety of cells in the lungs and is highly expressed in type II alveolar cells and macrophages in patients with pulmonary fibrosis (38). CD147 can also interact directly with the RBD domain of SARS-CoV-2 S protein because humanized anti-CD147 antibody can competitively inhibit the binding of S protein and CD147 and inhibit virus infection in host cells, suggesting that CD147 may also be one of the receptors mediating SARS-CoV-2 infection (39). This finding has been recognized by other research groups, and through determination that the binding affinity of CD147 to SARS-CoV-2 S protein is 0.185 uM, which is 15 nM lower than the affinity of ACE2 to S protein (40).

Also, Type II transmembrane glycoprotein C-type lectin dendritic cells specifically bind to non-integrated molecules (Dendritic cell-specific ICAM-3 grabbing non-integrin, DC-SIGN) and liver/lymphocytes specifically bind to non-integrated molecules (Liver/lymph cell-specific ICAM-3 grabbing non-integrin, L-SIGN) are reported to interact with SARS-CoV S protein to mediate virus invasion, but its mediated infection efficiency is much lower than that of ACE2. L-SIGN is a potential receptor for SARS-CoV, similar to Ebola virus and Sindbis virus. In addition, L-SIGN can also internalize viruses and promote virus degradation in a proteasome-dependent manner (41). The three potential receptors of the SARS-CoV-2, ACE-2, DC-SIGN, and L-SIGN, have higher expression levels in the lungs of smokers and the elderly, and higher expression levels in whites than Asians. However, this study is only based on gene expression databases, and more experiments are needed for verification (42).

Studies have confirmed that SARS-CoV-2 S protein can bind to neuropilin-1(NRP-1) on the surface of host cells through the CendR domain of the S1 protein subunit. The interaction of S1 CendR-Neuropilin-1 may promote the invasion and infection of SARS-CoV-2 (43, 44). Additionally, ASGR1 is an endocytic recycling receptor, which plays a key role in serum glycoprotein homeostasis and is reported to promote the invasion of hepatitis C virus (45, 46). Wnt/β-catenin signal transduction is essential for taste bud cell renewal and behavioral taste perception, and KREMEN1 is a negative regulator of this pathway, which can antagonize classic WNT signal transduction, and is also an invasion receptor for most enteroviruses. Odor and taste loss is often observed in COVID-19 patients, which suggests that SARS-CoV-2 may act through these receptors, thereby affecting Wnt/β-catenin signaling and causing loss of taste (47, 48). ASGR1 and KREMEN1 are speculated to be the co-receptors of SARS-CoV-2. Studies show that the susceptibility to viruses in airway epithelial cilia, secretory cells and immune macrophages are highly correlated with the expression of ACE2, KREMEN1 and ASGR1, respectively, and ACE2/ASGR1/KREMEN1 (ASK) together show a greater correlation than any single one (49). The interaction between the virus and the host receptor can induce cytokine secretion, apoptosis and stimulate immune response. Both LILRB2 and SIGLEC-9 are mainly expressed in myeloid cells, and COVID-19 is related to the excessive activation of myeloid cells. Therefore, these receptors may be involved in the activation of pro-inflammatory monocyte-derived macrophages, which in turn causes local inflammation (Figure 1) (49, 50).

In addition to the receptor, the host protease acting on the S protein can also promote viral infection. After the S1 subunit of the coronavirus S protein recognizes and binds the cell receptor, some proteases on the surface of the target cell will cut the S protein into S1 and S2 subunits, and then the S2 subunit will induce the fusion of the virus membrane and the cell membrane. If there are no these proteases on the cell surface, the virus will enter the host cell through endocytosis, and then the S protein will be cut into S1 and S2 subunits by cathepsin in endosome or lysosome, and then membrane fusion will occur to complete the invasion of the virus (51). Furin protease is a member of the precursor protein invertase (Proproteinconvertase, PACE) family, which mainly recognizes arginine-rich protein sites, can cleave secretory protein precursors into active proteins, and plays an important role in membrane receptor maturation, tumor metastasis, processing and activation of viral coat proteins and bacterial exotoxins. The existence of Furin protease cleavage site in SARS-CoV-2 S protein makes its infection mechanism different from that of most coronaviruses such as SARS-CoV, but more similar to that of HIV, Ebola virus and some avian influenza viruses, which may be one of the reasons why its transmission ability is higher than that of SARS virus CoV (52). Transmembrane prostease serine 2 (TMPRSS2) belongs to type II transmembrane protein, which is mainly located on the surface of cell membrane. It is highly expressed on the surface of respiratory epithelial cells and participates in the activation of many respiratory viruses. It can cleave the S proteins of coronavirus SARS-CoV, MERS-CoV, HCoV229 and SARS-CoV-2, and promote the fusion between virus and target cells. TMPRSS2 forms a receptor proteasome complex with ACE2, and cut the extracellular domain of receptor ACE2 to improve the efficiency of direct invasion of SARS CoV on the cell surface (53, 54). Transmembrane prostease serine 11a (TMPRSS11a), Human airway trypsin-like protease (HAT), Trypsin, Thermolysin and Elastase can induce the fusion of SARS-CoV and cell membrane on the cell surface and promote virus infection, so it is speculated that they may have similar functions for SARS-CoV-2 (55). Cathepsins include cysteine, serine, and aspartyl proteases with endopeptidase and exopeptidase activities. They are widely distributed in the endosomes and lysosomes in the acidic environment of cells, and play a role in degrading proteins and processing antigens (56). Studies have proved that Cathepsin L (CatL) inhibitors is possibly safer and more effective therapy to block coronavirus host cell entry and intracellular replication, without compromising the immune system (57). In the SARS-CoV related study, some other factors are also involved in the process of virus entry. Interferon-induced transmembrane protein (IFITM) can inhibit the virus from entering the cell through the endocytic pathway (58) and Phosphatidylinositol-4-kinase IIIβ (PI4KB) can produce the lipid microenvironment needed to promote the virus to enter the cell when the S protein invades the cell (59).

In summary, host cell receptors are a key determinant of virus tropism and pathogenesis. In addition to ACE2, SARS-CoV-2 can also interact with multiple receptors to invade the human body. These receptors are likely to interact with SARS-CoV-2 under different environmental or physiological conditions, trigger different signals, and ultimately lead to virus infection and host immune response, thereby promoting the pathogenic process of the virus. In view of the fact that SARS-CoV-2 has a stronger spreading power than SARS-CoV and is more harmful, scientists around the world are conducting research on SARS-CoV-2 and more specific mechanisms of SARS-CoV-2 invading cells will be clarified.

## Clinical Features of COVID-19

SARS-CoV-2 targets the respiratory tract, but the initial manifestations associated with COVID-19 are not specific (Figure 1), so it is difficult to distinguish between COVID-19 and other coronavirus infections, including SARS and Middle East respiratory syndrome (MERS) (60–62). When patients present with acute respiratory infection signs, including fever, cough, and headache, at the beginning of the illness, it is easily misdiagnosed, indicating clinicians should pay much attention to the underlying causes.

COVID-19 can be asymptomatic, and it can lead to mild or severe disease, or even death. The incubation period for COVID-19 is generally <14 days, and the mean incubation period is ~5 days (63). However, Wang et al. collected clinical data of 2015 laboratory-confirmed COVID-19 patients, and found that the incubation period varied from 0 to 33 days, and the incubation period of 11.6% of the patients was longer than 14 days (64). Individuals at any age can be infected with SARS-CoV-2, and the average age of patients with COVID-19 is 49.8 years. The most predominant symptoms in mild COVID-19 patients are fever, cough, fatigue, myalgia, and dyspnea (65, 66). Gastrointestinal symptoms, including diarrhea, nausea, and vomiting, have also been reported, but were less common (63). However, the digestive symptoms could be preceding the onset of respiratory symptoms in mild COVID-19 patients (67). Interestingly, it was reported that sudden and complete loss of olfactory function is also a main symptom in patients with COVID-19 (68). A multicenter study revealed that olfactory and gustatory dysfunction, which are less common in Asia, could be significant symptoms in European patients with mild-to-moderate COVID-19 (69, 70). The significantly different manifestations in different regions remind us to highlight the complexity of COVID-19. In patients recovering from COVID-19, the hallmark of COVID-19 is the presence of ground-glass opacities (GGOs) in CT images of the lungs at the early stage after symptom onset (71). In the progressive stage (5–8 days after symptom onset), patients present with more GGOs, extending to multiple pulmonary lobes and with a crazier paving appearance. The CT score and the number of lung zones involved aggravate rapidly, peaking at ~10 days after symptom onset. At this stage, consolidation and diffused GGOs are predominant findings. After 2 weeks, lesions are gradually absorbed, with a decreased GGO ratio (72, 73). Patients with severe COVID-19 are more likely to experience severe complications, including acute cardiac injury, arrhythmia, acute kidney injury, and shock (70). In terms of laboratory findings, an increased erythrocyte sedimentation rate (ESR) and elevated levels of C-reactive protein (CRP), lactate dehydrogenase (LDH), ferritin, interleukin 6 (IL-6), and tumor necrosis factor-α (TNF-α) are commonly observed in patients with COVID-19. The lymphocyte count is generally below normal values (74). Recently, high rate of thromboembolism has also been defined as an important feature of patients with COVID-19 (75, 76). Compared with mild patients, higher levels of plasma cytokines were observed among severe patients, suggesting an immunopathological process caused by a cytokine storm (77, 78). Fortunately, most patients recovered enough to be discharged in 2 weeks.

## Risk Factors for Severe Disease and Death

Patients older than 80 years old have a substantially higher CFR than younger patients (79). Severe infection was found to be associated with older age and male gender (80). It was reported that race could be identified as an independent factor for severity and death in COVID-19 patients. Black and other minority races were associated with higher risk of hospitalization as well as severity and mortality (81, 82). Patients having any medical comorbidities (e.g., obesity, diabetes, tumor, or heart, lung, or kidney diseases) have a greater risk of developing severe COVID-19 and higher mortality rates (70, 83). The ESR and levels of CPR, IL-6, LDH, high-sensitivity cardiac troponin I, N-terminal prob-type natriuretic peptide, creatine kinase, D-dimer, ferritin, creatinine, liver enzymes, and procalcitonin are commonly high in severe COVID-19 cases. Smoking, lymphopenia, low serum albumin levels, longer prothrombin time, and oxygen saturation <88% are also associated with the severity of COVID-19 (84, 85). Pregnancy was also a risk factor for severe infection and death caused by SARS-CoV-2 (86). The data revealed a higher viral load of nasopharyngeal swabs at the time of admission and a longer virus shedding period in patients with severe COVID-19 than in patients with non-severe COVID-19 (87). However, this relationship was not observed in posterior oropharyngeal and throat swab samples in different studies (88). However, the patient with the longest duration of virus shedding (49 days) had mild infection and a favorable outcome (89). Thus, the association between virus dynamics and severity of COVID-19 require further study. Although neutralizing antibodies were detected in all patients, the antibody titer showed no close correlation with the clinical course (90). Blood group A is associated with a higher risk of COVID-19 mortality compared with non-A groups, with an OR of 1.482 (95% CI, 1.113–1.972). Conversely, group O individuals had a lower risk of death (91). Compared with Rh+ blood type, Rh- was protective against SARS-CoV-2 infection and was associated with lower risk of severe COVID-19 illness or death (92). Environment is another important factor affecting COVID-19 mortality; COVID-19-induced death is positively associated with the diurnal temperature range and negatively with temperature and humidity (93). Recently, SARS-CoV-2 variant of concern B1.1.7 (VOC) has been identified in United Kingdom (UK). Infection with VOC was associated with higher rate of death (94). Hu et al. performed genome-wide association study and eight genetic variants were identified to significantly increase the risk of COVID-19 mortality (95), suggesting the genetic basis of heterogeneous susceptibility.

## Transmission Models

**Figure 2:**
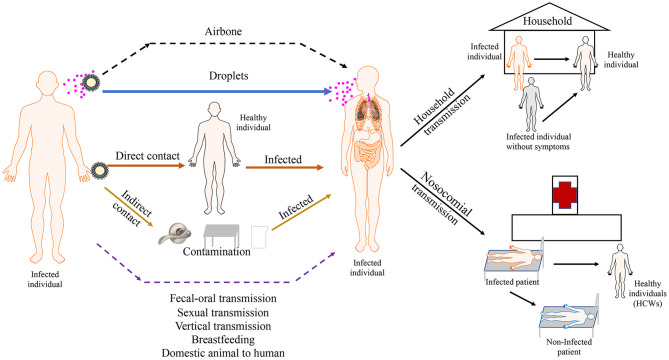
Transmission routes of SARS-COV-2. SARS-CoV-2 predominantly spreads via droplet transmission and through direct contact. the main transmission route is droplet transmission. The persistence of SARS-CoV-2 on inanimate surfaces (Fomites transmission) is likely a compounding factor for viral transmission. Airbone, fecal-oral, sexual and vertical transmission haven't been observed in current cases. Viral transmission through breastfeeding and transmission from domestic animal to human still need to be confirmed. The main transmission of SARS-CoV-2 occurs between family members. Nosocomial transmission is supposed to be an important route of infection. Asymptomatic and pre-symptomatic individuals appear to be a substantial threaten for public health because of high secondary attack rate of them. Solid arrows show confirmed transmission route. Dashed lines show possible transmission routes needed to be confirmed.

SARS-CoV-2 RNA is commonly detected in clinical specimens from different sites of COVID-19 patients, including bronchoalveolar lavage fluid, lung tissue, endotracheal aspirates, blood, serum, sputum, feces, rectum and anal swabs, and oral and throat swabs (96, 97). It is sometimes identified in urine samples (96). SARS-CoV-2 predominantly spreads via the respiratory tract and through close contact (2, 98), and the main transmission route is droplet transmission (99) (Figure 2). When infected patients cough, sneeze, or talk, viruses are released from the respiratory tract, which are able to infect people within 2 m proximity by directly infecting the mucous membranes. Until now, evidences for possible airborne transmission of SARS-CoV-2 are still accumulating. It was reported that SARS-CoV-2 was detected in air samples from newly built hospital with a concentration up to 42 copies/m^3^ (100). Airborne transmission has not yet been reported. A case of nosocomial transmission from two patients to healthcare workers (HCWs) is reported and the result addresses that a majority transmission of SARS-CoV-2 is close contact, rather than airborne route (101). However, SARS-CoV-2 is detectable in aerosols for up to 3 h, providing the possibility of aerosol transmission for SARS-CoV-2 in confined spaces or under specific circumstances (102). In addition, SARS-CoV-2 is stable on copper, cardboard, plastic, and stainless steel for several hours to days, suggesting that people can be infected if they touch their nose, mouth, or eyes after contacting contaminated objects (102). Although SARS-CoV-2 RNA could be detected in the conjunctival swab samples of COVID-19 patients and some COVID-19 patients had ocular manifestations, such as conjunctivitis, transmission through the conjunctival route does not seem probable because of the limited number of clinical cases (103). Nevertheless, it is necessary for HCWs to protect their eyes. Recently, SARS-CoV-2 RNA was detected in gastrointestinal tissues, and the successful isolation of SARS-CoV-2 from stool samples demonstrated the possibility of fecal–oral transmission, although there is a lack of direct evidence and no clinical cases have been reported (96, 104). Some studies report that semen samples, testicular tissues, and the vaginal environment of COVID-19 patients are negative for SARS-CoV-2 RNA, supporting the notion that SARS-CoV-2 is not sexually transmitted (105). However, recent study finds that SARS-CoV-2 is detectable in semen samples of COVID-19 patients (6 of 38 patients) and 2 of these patients have achieved clinical recovery (106). The result provides the possibility that SARS-CoV-2 can be transmitted sexually, although no clinical evidence supported sexual transmission. A study finds that SARS-CoV-2 RNA can be detected in placental and fetal membrane sample, however, none of infants tested positive for SARS-CoV-2 in the first 5 days of life (107). In another study, seven neonates are tested within the first 24–36 h of life and only one has positive result (108). Although a neonate was diagnosed with COVID-19 36 h after birth, SARS-CoV-2 RNA was not detected in the placenta and cord blood (109). Similarly, a newborn with COVID-19 was successfully delivered, although SARS-CoV-2 RNA was not detected by PCR in the amniotic fluid, throat swabs, and rectal swabs at birth and neonatal blood was negative for antibodies (IgM and IgG) against SARS-CoV-2 (110). However, elevated levels of antibodies (IgM and IgG) against SARS-CoV-2 were observed in another neonate 2 h after birth. Interestingly, multiple PCR results on nasopharyngeal swabs taken between 2 h and 16 days after birth were negative. Unfortunately, PCR analysis of the amniotic fluid or the placenta was not performed (110). Based on these clinical cases, we cannot determine whether COVID-19 can spread vertically. In addition, low risk of intrapartum SARS-CoV-2 transmission to the newborn is possible during vaginal delivery (111). Another major concern about pregnant COVID-19 patients is the safety of breastfeeding after childbirth. In several studies, the detection of SARS-CoV-2 RNA in breastmilk was negative (110). Recently, SARS-CoV-2 is detectable in one patient's breastmilk. Additionally, even after delivery for 2 and 3 days, the results remain positive (112), which highlights the concern of SARS-CoV-2 infection risk through breastfeeding. Several studies have indicated that SARS-CoV-2 can infect domestic animals, including cats and dogs (23, 24). It is reported the virus is transmissible between cats and ferrets (113, 114). Although no cases of transmission from domestic animals to humans were confirmed, animal to human transmission can't be ignore. Munnink et al. provided evidence of mink to human transmission of SARS-CoV-2 within mink farms (21). The full picture of transmission routes is not yet complete (Figure 2), so additional studies are necessary before including or ruling out certain routes.

## Risk Factors for Human-to-Human Transmission

Human-to-human transmission has been reported within community, household, and nosocomial settings. According to current clinical data, individuals of all ages are susceptible to SARS-CoV-2 infection. However, people in close contact with confirmed COVID-19 patients are at a higher risk of infection. A recent study included 4,950 persons having close contact with confirmed COVID-19 patients. After 14 days of isolation or after symptom onset, throat swabs were collected for PCR analysis. Older age was significantly associated with an increased risk of infection (*P* = 0.0016). However, this association was not observed in another study (115). Hence, it remains unknown whether age is a risk factor of infection for people in close contact with confirmed COVID-19 patients. Household contact (including sharing a room, apartment, or other sleeping arrangements) (OR, 6.3; 95% CI, 1.5–26.3) is more likely to pass the infection (115, 116). Patients with severe infection have a higher risk of spreading the virus to close contacts (116). Another study showed that sharing a bedroom and being spoken to by an index case for 30 min or longer were identified as risk factors of viral transmission among household contacts (117). HCWs working at the forefront to fight COVID-19 run the greatest risk of getting infected. HCWs in the respiratory department, infection department, intensive care unit (ICU), and surgical department are particularly vulnerable to SARS-CoV-2 infection. HCWs work long hours and have suboptimal hand hygiene after contacting COVID-19 patients, and they are therefore at a high risk of SARS-CoV-2 infection (118). If the number of encounters and the time of interaction between HCWs and COVID-19 patients increase, the chance of HCWs getting infected also increases. A crowded workplace likewise increases the risk to HCWs of infection by SARS-CoV-2. Personal protective equipment (PPE) significantly decreases the infection risk for HCWs (119). High-risk occupations also include drivers, transport workers, and sales workers (120). The potential risk of COVID-19 transmission among hospitalized psychotic patients should not be ignored, especially those without insight (121). In addition, Zhao et al. analyzed the association of ABO blood type and the susceptibility to COVID-19 in 1,775 patients infected by SARS-CoV-2; they found the population with blood group A had a significantly higher risk of SARS-CoV-2 infection (OR, 1.279; 95% CI, 1.136–1.440). The population with blood group O had a significantly lower risk of SARS-CoV-2 infection (OR, 0.680; 95% CI, 0.599–0.771) (91). The environment is also a major factor influencing the incidence of SARS-CoV-2 infection. Inactivation of SARS-CoV-2 on surfaces will be accelerated by increasing temperature and relative humidity (122). These identified risk factors allow managers to take effective measures to prevent and control the transmission of SARS-CoV-2 in the early stage.

## Household Transmission

The main transmission of SARS-CoV-2 occurs between family members, including relatives and their friends, who may intimately contact carriers or patients (Figure 2). On January 10, 2020, Chan et al. enrolled a family of six patients, all infected with SARS-CoV-2 (123). Five of them, aged between 36 and 66 years, presented with fever and upper or lower respiratory disease symptoms. Another child, aged 10 years, was asymptomatic with radiological pulmonary GGOs. They found that patients aged >60 years had more systemic symptoms, such as extensive radiological pulmonary ground-glass changes and lymphopenia, compared with the child. On February 4, 2020, Ye et al. reported a person who was infected with SARS-CoV-2, and her family members were admitted to the hospital the next day (124). In this family-clustered SARS-CoV-2 infection case, the first patient who got pneumonia had only had contact with her four family members, and the day when she attended the family reunion dinner, she did not have any SARS-CoV-2 symptoms. Li et al. reported a patient who was infected with SARS-CoV-2 and had lived together with other families for 4 days when he did not have any symptoms (125). However, several days later, one of his relatives who had lived with him previously was diagnosed with SARS-CoV-2 infection. The next day, as the closest contact of that relative, he was confirmed to have SARS-CoV-2 infection by PCR of his swab samples. These observations indicate SARS-CoV-2 is highly infectious and might be transmitted by asymptomatic carriers during the incubation period. The infectivity during this incubation period in the household is a big challenge for disease control. Recently, the characteristic of household transmission is assessed. Wang et al. enrolled 85 patients and their household members, and all close contacts received RT-PCR analysis. They found the rate of secondary transmission among household contacts of COVID-19 patients was 30% (126). In another study, 27,101 households with 29,578 primary cases and 57,581 household contacts were enrolled. The estimated secondary attack rate within households was 15.6% and older individuals were more susceptible to SARS-CoV-2 infection (127). Reukers et al. enrolled a total of 55 households with 187 household contacts in Dutch. Estimated secondary attack rate was high and ranged from 35% in children and 51% in adults (128). These data reinforced the role of households as a major of transmission route of SARS-CoV-2 infection.

## Nosocomial Transmission

Given the high infectivity of SARS-CoV-2, hospital-related transmission is supposed to be an important route of infection (Figure 2). Wang et al. reported hospital cases of COVID-19 (74). Of the 138 patients in their report, 57 (41.3%) were presumed to be infected with SARS-CoV-2 in the hospital, including 17 patients (12.3%) who were hospitalized previously for other reasons and 40 HCWs (29%). One patient who had SARS-CoV-2 and presented with abdominal symptoms was soon admitted to the surgical department, and more than 10 HCWs who worked in the surgical department were presumed to be infected by this patient. This report also revealed that patient-to-patient transmission seems to occur. At least four hospitalized patients in the same ward as an infected patient were infected; those infected patients all presented with atypical abdominal symptoms, and soon one of the four patients was diagnosed with COVID-19 during his hospitalization. Characteristics of HCWs who underwent SARS-CoV-2 testing in Italy were described. There were 139 positive result among 1,573 HCWs. Among infected HCWs, 122 patients were symptomatic and 17 patients were asymptomatic. The highest frequency of infection was occurred in physicians, while clerical workers and technicians were the groups with the lowest infection rates. The key symptoms for symptomatic HCWs to guide diagnosis were taste and smell disorders. A median of 27 days is necessary from first positive test to a negative test (129). Fortunately, there wasn't significant association between nosocomial COVID-19 and increased mortality (130). Notably, various studies have proposed that there are multiple possible routes of in-hospital transmission. A retrospective study including 66 hospital-acquired cases demonstrated that evidence of transmission through close contact was found in 55% cases. Cross-infection may have occurred in 14% cases through using shared facilities and equipment. However, no sources were identified for the remaining cases (131). Goldberg et al. firstly reported 6 HCWs were infected from 1 family despite using PPE and keeping physical distance, providing possible evidence for airborne transmission of SARS-CoV-2 (132). Therefore, comprehensive infection prevention and control measures should be performed in hospital.

## SARS-CoV-2 Transmission Without Symptoms

Asymptomatic patients include those with positive nucleic acid test yet without clinical symptoms, whereas pre-symptomatic individuals develop symptoms later in the course of infection. When some experts believe that because patients without symptoms have no cough, sneeze, or other clinical symptoms, the chance of transmission caused by pathogenic discharge from the body is smaller than that of confirmed cases, while some think that risk of transmission rates of asymptomatic or pre-symptomatic patients should not be underestimated (Figure 2). Since the viral load in the respiratory tract samples of asymptomatic patients is not significantly different from that of confirmed cases (133). Another report suggested that patients were infectious 1–3 days before any symptoms appeared (134). Moreover, a typical cluster spreading event was reported where an asymptomatic person went to a public bath center and transmitted SARS-CoV-2 to eight other individuals (135). Transmission among family clusters was also observed in a hospital in Beijing after the nephew of index patients was found to be SARS-CoV-2-positive by PCR assay (136). This study also showed that asymptomatic individuals can transmit SARS-CoV-2. In China, a large SARS-CoV-2 outbreak caused by a single asymptomatic individual was reported in Heilongjiang Province and a total of 71 positive cases had been identified (137). Wu et al. enrolled 185 asymptomatic cases with 1,078 close contacts. They found the secondary attack rate among close contacts of asymptomatic cases was 1.1%, which was lower than that of symptomatic cases (4.1%). More than one third of the infections occurred from exposure to symptomatic cases was attributed to pre-symptomatic transmission. In addition, infected contacts of asymptomatic cases were less likely to be severe (138). Compared with asymptomatic transmission, pre-symptomatic transmission was more likely to have a higher secondary attack rates (139). Although asymptomatic transmission posed a lower transmission risk, they still appeared to be a substantial threaten for public health. For pre-symptomatic transmission, it was confirmed that viral shedding is higher before symptoms begin. Therefore, it was critical to ensure individuals exposed to confirmed patients stay home.

## SARS-CoV-2 Viral Load and Transmission

The viral loads detected in asymptomatic patients are similar to those detected in symptomatic patients, indicating the transmission potential of asymptomatic and mild patients are similar (133). Rapid viral proliferation was detected 0–5 days before symptom onset and highest viral load in throat swabs was observed at the time of symptom development (140, 141). In addition, 44% of secondary cases were infected due to pre-symptomatic transmission, indicating there was a positive association between viral load and transmissibility of SARS-CoV-2 (141). Compared with ono-index patients, viral load at the initial sample collection was higher in the index patients (142). Mark et al. identified 314 COVID-19 patients with 753 contacts in total. They found the secondary attack rate when the index cases had a viral load lower than 1 × 10^6^ copies per mL was 12%. However, when the viral load of the index cases was higher than 1 × 10^10^ copies per mL, the secondary attack rate was 24% (143). Goyal et al. identified the secondary attack rate was very low when the viral load of infected person was lower than 1 × 10^4^ copies per mL. On the other hand, transmission was much more likely (39%) when the infected person was shedding > 1 × 10^7^ copies per mL, and 75% when the viral load was higher than 1 × 10^8^ copies per mL. Furthermore, Massive super-spreading events always occurred at viral loads exceeding 1 × 10^7^ copies per mL (144). These data demonstrated that the viral load of index cases was a strong drive of SARS-CoV-2 transmission.

## Fomites Transmission and SARS-CoV-2

Various studies suggested persistence of SARS-CoV-2 on inanimate surfaces for days, with potential implications for viral transmission. In one study, the distribution of SARS-CoV-2 in hospital wards was examined by testing air and surface samples. The results revealed that contamination in ICUs was more widespread than in general wards. Viruses were widely distributed on garbage cans, computer mouses, floors, and sickbed handrails, and were detected in air ~4 m away from patients (145). Extensive environmental contamination has been detected, and toilet bowl and sink samples of SARS-CoV-2-infected patients were tested positive (146). Liu et al. measured viral RNA in aerosols in different areas in two hospitals. They found that the concentration of SARS-CoV-2 RNA in aerosols was higher in the toilet areas used by the patients. Airborne SARS-CoV-2 was detectable in two crowd areas, while levels of airborne SARS-CoV-2 couldn't be detected in the most public areas. High concentration of viral RNA also could be detected in some medical staff areas (100). Of the 182 isolation ward samples, SARS-CoV-2 RNA was detectable in 9 samples. These positive samples were collected from a facemask, the floor, mobile phones and the air in the patient room and bathroom (147). Therefore, fomite transmission may occur indirectly through touching infected surfaces or objects. Xie et al. reported an evidence of indirect transmission of SARS-CoV-2 (148). They found individual could be infected through touching an elevator button contaminated by index patients (148). Although the fomite transmission is difficult to prove definitively, we also need to take some measure to prevent it. Hand hygiene is a barrier to fomite transmission and is closely associated with lower risk of infection (149).

## Mutant Strains of SARS-CoV-2 and Transmission

Recently, several novel variants strains of the SARS-CoV-2 virus have emerged carrying multiple mutation during the COVID-19 pandemic. SARS-CoV-2 B.1.1.7 was first identified in September 2020 in England, while B.1.351 was detected in late 2020 in South Africa (150). In addition, B.1.1.28.1 (P.1) variant and CAL.20C (B.1.427/B.1.429) were first detected in Brazil and California, respectively (151, 152). A major concern about these mutation strains is whether any of these variants have the ability to alter viral features, such as the transmission mode or rate. These variants share a special point mutation, named D614G. D614G, a non-synonymous mutation, results in a replacement of aspartic acid with glycine at position 614 of the virus's spike protein (153). Using multiple human cell lines, Daniloski et al. found that D614G variant was more effective at entering cells (154). Hou et al. generated SARS-CoV-2 variant harboring mutation D614G and explored the effects of D614G on SARS-CoV-2 infectivity, spread and transmission (155). Compared with D614 virus, G614 virus have higher efficiency to enter cell lines and D614G substitution enhances SARS-CoV-2 replication fitness in the primary cells. Importantly, G614 virus transmitted faster between hamsters than the D614 virus, suggesting D614G variant may confer increased transmissibility (155). Similar results were observed in Zhou's study. They found D614G substitution enhanced the ability to bind to ACE2 and also increased replication in primary human epithelial cells and hACE2 knock-in mice model. Notably, increased transmissibility of D614G substitution was also observed in ferret models of SARS-CoV-2 infection (156). Zhao et al. analyzed that a per 0.01 increase in the prevalence of D614G substitution was associated with a 0.49% increase in time-varying reproduction number, indicating a significant positive association between COVID-19 transmissibility and the D614G substitution (157). In addition to the mutation caused the D614G substitution, these new mutation strains also contain other different mutations in the spike gene, including N501Y substitution, E484K substitution, K417N substitution, L452R substitution and deletion mutations (ΔH69/ΔV70 and ΔY144) (158). Therefore, there is a growing concern that new variants could alter the transmission model and rate of the virus. Using a variety of statistical and dynamic modeling approaches, Davies et al. estimated that B.1.1.7 variants has a 43–90% higher reproduction number than original variants and will result in large resurgences of COVID-19 cases. Concerningly, B.1.1.7 has emerged globally and shows an increased transmission rate in many countries (159). By sequencing and analyzing B.1.1.7 SARS-CoV-2 genomes, Washington et al. found that growth rate of B.1.1.7 was at least 35–45% increased and doubling every week and a half in USA (160). Using globally available data, Pearson et al. assessed the B.1.351 variant for increased transmissibility and estimated that B.1.351 was 1.50 times as transmissible as previous variants (161). B.1.427/B.1.429 variants emerged around May 2020 and dramatically increased from September 2020 to January 2021, demonstrating an 18.6–24% increase in transmissibility compared with original strain (162). Faria et al. integrated available data by employing two-category dynamical model and estimated that P.1 may be 1.4-2.2 times more transmissible than non-P.1 lineages (163). Notably, B.1.617 (double mutant) has been reportedly in India and caused a rapidly increased COVID-19 cases in the country. Three primary mutations have been detected in spike glycoprotein in B.1.617 lineage, including P681R, E484Q, and L452R-the latter two mutations were mutation of concern (164). Therefore, many people also have referred to the variant as the “double mutant.” In fact, the two mutations have been found in other variants separately. L452R has been spotted in B.1.427/B.1.429 variants, while E484Q was similar to the E484K detected in the B.1.351 and B.1.1.28.1. It was the first time that these two mutations were reported to coexist together, which was an indication of higher transmissibility. Compared with wild type, E484Q and L452R mutants in B.1.617 lineage demonstrated an increased hydrogen bond interaction with hACE2. In addition, B.1.617 mediated an enhanced entry into the human lung and intestine-derived cell lines Calu-3 and Caco-2, respectively, suggesting a higher transmissibility (165, 166). Compared with B.1 (D614G) variant, the increased severity of B.1.617 infection in hamsters was evident by the higher viral load and body weight reduction, and more sever lung lesions (167). L452R was previously demonstrated to be resistant to some neutralizing antibodies (168). E484K mutation also conferred antibody resistance (169). It was found B.1.617 evaded antibodies induced by infection and vaccination, although with moderate efficiency, which might contribute to increased transmission dynamics (166, 170).

## SARS-CoV-2 Vaccine

A safe and effective vaccine against SARS-CoV-2 will be an important tool to control the global COVID-19 pandemic. BNT162b2, a nucleoside-modified RNA vaccine, encodes the SARS-COV-2 full-length spike and is modified by two proline mutations to lock it in the prefusion conformation (35). The findings from the phase 2/3 part of a global phase 1/2/3 trial revealed that BNT162b2 conferred 95% protection against COVID-19 infection in persons 16 years of age or older from 7 days after the second dose (30 ug/dose, given 21 days apart). There wasn't significant difference in incidence of serious adverse events between placebo and vaccine group (171). In addition, BNT162b2 also was 51% efficient in preventing SARS-CoV-2 infection 13–24 days after the first dose (172). A real-world evidence confirmed that viral load was significantly decreased for infections occurring 12–27 days after the first dose, suggesting a lower infectiousness (173). Antibody responses induced by the first dose of BNT162b2 in individuals with prior infection were similar to those seen after a two-dose vaccination in individuals without prior infection, suggesting a single dose of BNT162b2 is sufficient for previously infected individuals. After the first dose, symptomology was more prominent for individuals with prior infection. Post-vaccine symptoms were similar between groups after second dose (174). Recently, several variants of SARS-CoV-2 have emerged and it was unclear whether these new strains could be neutralized efficiently by BNT162b2. For B.1.1.7 lineage, Slight reduction of the immune sera was observed. However, the overall largely preserved neutralization of B.1.1.7 lineage by BNT162b2-immune sera made it unlikely to escape from BNT162b2-mediated protection (175). Sasone et al. analyzed the evaluated the effectiveness of BNT162b2 in Brescia country, where the B.1.1.7 variant was highly prevalent (70–97%). They found the vaccine was effective in reducing infection rate among vaccinated HCWs (176). The estimated effectiveness of the BNT162b2 against infection with B.1.1.7 variant and B.1.351 variant was 89.5 and 75%, respectively. BNT162b2 against severe, critical or fatal infection with B.1.1.7 or B.1.351 variant was 97.4% Although vaccine effectiveness against B.1.351 variant was 20% lower than the effectiveness against original virus, it was still highly effective against severe and critical cases (177). Similar results were observed in another studies (178, 179). In addition, Xie et al. found neutralization geometric mean titers of human sera elicited by BNT162b2 against N501Y, 69/70-deletion+N501Y+D614G and E484K+ N501Y+D614G mutant virus were 0.81–1.46-folds of the geometric mean titers against wild-type strain, indicating small effects of these mutations on neutralization by BNT162b2 vaccine-elicited sera (180). A phase 3 trial reported that mRNA-1273 vaccine was 94.1% efficient in preventing COVID-19 illness from 14 days after the second dose and no safety concerns were identified (181). Similarly, B.1.1.7 variant escapes a subset of monoclonal antibodies but remains susceptible to neutralizing antibodies elicited by mRNA-1273 vaccine (182). The protective humoral immunity induced by mRNA-1273 vaccine was still retained against B.1.351 variant (183) and B.1.429 variant (184). One of the candidate vaccines Ad26.COV2.S, a recombinant adenovirus serotype 26 vector encodes the spike glycoprotein (185). On April 13, 2021, FDA had recommended a pause in the use of Ad26.COV2.S vaccine due to the reports of a rare and severe type of blood clot (186). CoronaVac, an inactivated vaccine candidate against SARS-CoV-2, developed by Sinovac Life Science (Beijing, China). The phase 3 clinical trial confirmed that CoronaVac was 50.7% efficient in preventing SARS-CoV-2 infection, 83.7% efficient in preventing moderate cases and 100% efficient in preventing severe cases. In addition, B.1.128, P.1 and P.2 variants were susceptible to neutralizing antibodies elicited by CoronaVac vaccine (187). BBIBP-CorV, another inactivated vaccine, was safe and well-tolerated. Immune responses could be induced rapidly and against SARS-CoV-2 (188). 501Y.V2 variant can't escape the immunity induced by BBIBP-CorV vaccine (189). In the context of the current, SARS-CoV-2 vaccine can contribute to reducing the infection rate and the devastating loss of health and life, together with other public health measures. Although slight reduction of the immune sera was observed, the overall largely preserved neutralization of SARS-CoV-2 variants made them unlikely to escape from vaccine-mediated protection. Furthermore, we still need to get more evidence in the real word.

## Recommendations

The cumulative number of confirmed cases of COVID-19 around the world is still increasing. The epidemic situation in some countries has reversed, virus mutations have occurred frequently, and the pressure on foreign defense imports has increased. In order to further guide the prevention and control of new coronavirus pneumonia in various regions, we give the following suggestions. (1) Everyone should maintain social distance, minimize indoor activities or control the number of participants. In principle, we need to ensure that the distance between people is more than 1 m, and all participants should strictly take personal protective measures, wear masks, and strictly conduct hand disinfection. (2) At present, the rapid detection technology of viral nucleic acid and serum has matured, and countries should strengthen the monitoring of people, objects and the environment, such as imported cold chain foods and items, and timely detect and report the epidemic situation. (3) Pay attention to pathogen monitoring, dynamically monitor virus mutations, and understand the impact of virus mutations on pathogen detection and vaccine protection. (4) Strengthen the management of quarantine medical observation for entry personnel and close contacts, and two nasopharyngeal swab samples should be collected at the same time when the quarantine is released, and different nucleic acid detection reagents should be used for testing. (5) Nucleic acid tests should be carried out on the 2nd and 7th days after the quarantine is lifted and health monitoring should be carried out to reduce mobility during the period. Individuals should maintain self-protection when going out and no gathering activities are allowed. (6) Once symptoms such as fever and cough occur, patients should wear a disposable medical surgical mask and try to walk or go to the hospital by private car. In the hospital, the corresponding treatment strategy is adopted according to the classification of the disease. (7) Improve the vaccination work for high-risk groups, and further improve the vaccination strategy based on the progress of vaccine research and development and clinical trial results. (8) Countries all over the world should introduce measures to prohibit the hunting, trading, and consumption of wild animals. This is for the sake of protecting human health and maintaining ecological harmony. (9) In view of the objective situation of relatively weak epidemic prevention and control capabilities in economically underdeveloped areas, the state and the government should focus on providing guidance and support for the epidemiological investigation, isolation medical observation, nucleic acid testing and disinfection. (10) According to the current situation of epidemic prevention and control, we need to conduct training on prevention, control, diagnosis and treatment plans for medical institutions. Improve the ability of medical and health personnel to ensure that the hospital finds suspicious cases and conducts isolation treatment as soon as possible, so as to effectively control the current epidemic situation in time.

## Conclusion

As an ongoing public health risk, SARS-CoV-2 has received much attention. Many studies support the idea that bats are the original host of SARS-CoV-2, and some animals, including minks, pangolins, snakes, and turtles, might be intermediate hosts, facilitating the primary transmission of SARS-CoV-2. To limit transmission to animals or humans, we must elucidate the evolutionary path from the original host to cross-species transmission. Respiratory infection signs are the predominant manifestations among COVID-19 patients, and extrapulmonary symptoms preceding the onset of respiratory symptoms should not be ignored. Clinicians should evaluate COVID-19 patients according to risk factors that are closely associated with the severity of COVID-19 for prioritized and aggressive treatment. Since there is no specific treatment, it is still a top priority to (i) control the spread of the virus, (ii) study the transmission routes, and (iii) identify factors that stimulate viral spread. (iv) get vaccination against SARS-CoV-2. Considering the threat posed by COVID-19, it remains a priority to strengthen international collaborative relationships to advance our knowledge of SARS-CoV-2.

## Author Contributions

TW, SK, WP, CZ, YZ, LP, KF, YY, XY, XL, LJ, and MD contribute to the material collection and manuscript writing. All authors read and approved the final manuscript.

## Conflict of Interest

XL was employed by Hunan Yuanpin Cell Biotechnology Co., Ltd. The remaining authors declare that the research was conducted in the absence of any commercial or financial relationships that could be construed as a potential conflict of interest.
